# The clinical features and prognosis of 100 AIDS-related lymphoma cases

**DOI:** 10.1038/s41598-019-41869-9

**Published:** 2019-03-29

**Authors:** Dedong Wu, Chen Chen, Mingzhi Zhang, Zhaoming Li, Suqian Wang, Jijing Shi, Yu Zhang, Dingzhu Yao, Shuang Hu

**Affiliations:** 1 Department of Medical Oncology, The First People’s Hospital of Zhengzhou, Zhengzhou, Henan 450004 China; 2 Department of Medical Oncology, The First Affiliated Hospital of Zhengzhou University, Zhengzhou, Henan 450052 China; 3 Personnel Section, The First People’s Hospital of Zhengzhou, Zhengzhou, Henan 450004 China; 4 Central Lab at Zhengzhou First People’s Hospital, The First People’s Hospital of Zhengzhou, Zhengzhou, Henan 450004 China; 5 Medical Records Room, Henan Provincial Infectious Disease Hospital, Zhengzhou, Henan 450015 China; 6 Comprehensive Ward, The First People’s Hospital of Zhengzhou, Zhengzhou, Henan 450004 China; 7 Department of Medical Oncology, Henan Provincial Infectious Disease Hospital, Zhengzhou, Henan 450015 China

## Abstract

To improve outcomes and risk assessment, we systematically analyzed the clinical features of patients with acquired immunodeficiency syndrome (AIDS)-related lymphoma (ARL) and identified survival-associated factors. Data were collected from 100 patients diagnosed with ARL at the Henan Provincial Infectious Disease Hospital in China. The progression-free survival (PFS) duration and 2-year overall survival (OS) rate were determined. A multivariate analysis was used to evaluate the associations between survival and the following variables: sex, age, histological subtype, Ann Arbor stage, lactate dehydrogenase (LDH) level, primary site, baseline CD4^+^ count, use of chemotherapy, and age-adjusted international prognostic index IPI (aaIPI). The timing of combined antiretroviral therapy (cART) relative to chemotherapy was also assessed. The PFS duration and 2-year OS rate were significantly higher in the chemotherapy vs. the non-chemotherapy group (P < 0.001), but did not differ significantly between patients who received chemotherapy before vs. simultaneously as cART (P > 0.05). Age, aaIPI, chemotherapy, LDH level, and the Burkitt/Burkitt-like lymphoma subtype were significant prognostic factors for 2-year OS; the other factors were not associated with prognosis. Our results show that cART plus chemotherapy significantly improves the survival of patients with ARL and identifies several prognostic factors.

## Introduction

Human immunodeficiency virus (HIV) infection increases the risk of malignancy. More than 28% of HIV-related deaths are attributed to malignant tumors, and more than 40% of HIV-infected people are eventually diagnosed with acquired immunodeficiency syndrome (AIDS)-related lymphoma (ARL)^1,2^. ARL is a rapid-growing and invasive malignancy and if untreated, may cause death within weeks or months after diagnosis. The introduction of combined antiretroviral therapy (cART, also known as highly active antiretroviral therapy) have led to significant decreases in the incidence of ARL and AIDS-related deaths^3^.

The pathology of ARL is heterogeneous. Diffuse large B-cell lymphoma (DLBCL) is the most common ARL subtype, accounting for approximately 45% of cases^4^. Burkitt lymphoma or Burkitt-like lymphoma (BL/BLL) is also common, followed by primary exudative lymphoma and oral plasmablastic lymphoma (PBL), whereas T cell lymphoma is rare^5^. Non-Hodgkin’s lymphoma (NHL) is the most frequent pathological subtype in patients with advanced HIV infection, a low CD4^+^ count (<100 cells/μL), and a high HIV load^2,6,7^.

Despite the reported decrease in incidence, ARL remains one of the most common causes of hospitalization for HIV-infected patients^8^. These patients depend on the selection of suitable treatments such as chemotherapy, cART, and targeted drug therapy, for which the efficacy and safety have been demonstrated. A combination of chemotherapy and targeted therapy has been approved for the treatment of CD20^+^ NHL, and the approved chemotherapy regimens include R-CHOP (rituximab plus cyclophosphamide, doxorubicin, vincristine, and prednisone) and R-EPOCH (rituximab, etoposide, prednisone, oncovin, cyclophosphamide, and hydroxydaunorubicin)^9^. In a UK study of DLBCL patients treated with rituximab-based regimens, patients with HIV infection had significantly higher 5-year overall survival (OS) (78% vs. 64%, P = 0.03) and disease-free survival rates (94% vs. 77%, P = 0.03), compared to those without HIV infection^10,11^. As rituximab has also been proven safe and well-tolerated, it has been approved for the treatment of B-cell lymphomas^12^.

Early studies of ARL reported associations of a poor prognosis with multiple factors, including patient-specific (age and performance status), HIV-specific (history of AIDS, and low CD4^+^ count), and lymphoma-specific factors (stage, lactate dehydrogenase [LDH] levels, extranodal disease, and a high international prognostic index [IPI])^13,14^. Patients with *Myc* rearrangements, EBV infection, and high-levels of NKp44/NCR2 often have a poor prognosis^15–19^. However, it remains unclear which prognostic factors are related to the prognosis of ARL.

In China, HIV-infected patients have traditionally been excluded from clinical trials due to poor prognosis or compliance. Accordingly, there are no HIV-specific guidelines for the management of ARL patients^20^. This retrospective study aimed to analyze the clinical features and outcomes of chemotherapy plus cART and the factors affecting prognosis in 100 ARL patients and thus provide information that would facilitate the establishment of such guidelines.

## Results

### Clinical characteristics of the study subjects

The characteristics of the 100 patients with ARL who were included in our analysis are shown in Table 1. The mean age was 43.6 years, and most patients were male (66%). DLBCL was the most common pathological type (66%), followed by BL/BLL (12%), Hodgkin’s lymphoma (6%), and other (16%). Fifteen patients received cART only, and 85 received cART plus chemotherapy. Forty-eight patients received cART before chemotherapy (prior cART group) and 37 received cART and chemotherapy simultaneously (concurrent cART group).

**Table 1:** Basic clinical features of the study population of patients with ARL.

Variables	All patients (n = 100)	NHL (n = 94)			HL (n = 6)
		DLBCL (n = 66)	BL/BLL (n = 12)	Other NHL (n = 16)	
Age (mean ± SD)	43.6 ± 13.0	42.7 ± 14.2	43.0 ± 13.4	49.4 ± 7.4	38.8 ± 4.7
**Sex, n (%)**					
Male	66 (66.0)	44 (66.7)	9 (75.0)	7 (43.8)	6 (100.0)
Female	34 (34.0)	22 (33.3)	3 (25.0)	9 (56.3)	0
**Baseline CD4 count, n (%)**					
Baseline CD4 < 100 cells/mm^3^	36 (36.0)	25 (37.9)	6 (50.0)	4 (25.0)	1 (16.7)
Baseline CD4 ≥ 100 cells/mm^3^	64 (64.0)	41 (62.1)	6 (50.0)	12 (75.0)	5 (83.3)
**Treatment, n (%)**					
Non-chemotherapy^a,*^	15 (15.0)	9 (13.6)	3 (25.0)	3 (18.8)	0
Chemotherapy^b,*^	85 (85.0)	57 (86.4)	9 (75.0)	13 (81.3)	6 (100.0)
Pre-cART^c^	48 (48.0)	36 (54.5)	3 (25.0)	7 (43.8)	2 (33.3)
Concurrent cART^d^	37 (37.0)	21 (31.8)	6 (50.0)	6 (37.5)	4 (66.7)
**Age-adjusted IPI, n (%)**					
1–2	34 (34.0)	26 (39.4)	0	3 (18.8)	5 (83.3)
3–4	66 (66.0)	40 (60.6)	12 (100.0)	13 (81.3)	1 (16.7)
**Ann Arbor stage, n (%)**					
Stage I–II	63 (63.0)	51 (77.3)	3 (25.0)	7 (43.8)	2 (33.3)
Stage III–IV	37 (37.0)	15 (22.7)	9 (75.0)	9 (56.3)	4 (66.7)
**LDH, n (%)**					
Normal LDH	32 (32.0)	18 (27.3)	3 (25.0)	7 (43.8)	4 (66.7)
Elevated LDH	68 (68.0)	48 (72.7)	9 (75.0)	9 (56.3)	2 (33.3)
**Primary site, n (%)**					
Intranodal	56 (56.0)	34 (51.5)	6 (50.0)	10 (62.5)	6 (100.0)
Extranodal	44 (44.0)	32 (48.5)	6 (50.0)	6 (37.5)	0

^a^Patients who received cART alone.

^b^Patients who received chemotherapy combined with cART regimen.

^c^Pre-cART: individuals who received cART treatment at least 3 months prior to chemotherapy.

^d^Concurrent cART: individuals who received cART treatment within 3 months before and after the first cycle chemotherapy.

^*^CHOP or EPOCH regimens were used for first-line chemotherapy in patients with non-Hodgkin’s lymphoma; ABVD regimens were used for first-line chemotherapy in patients with Hodgkin’s lymphoma.

DLBCL, diffuse large B-cell lymphoma; BL, Burkitt lymphoma; BLL, Burkitt-like lymphoma; IPI, International Prognostic Index; HL, Hodgkin’s lymphoma; Other NHL, Other NHL except DLBCL and BL/BLL. cART, combination antiretroviral therapy. LDH, lactate dehydrogenase.

Among the 100 patients in our study, 64 had ≥100 CD4^+^ cells/mm^3^, 66 had an aaIPI of 3–4, and 68 had an elevated LDH level. The primary lymphoma site was intranodal in 56 cases and extranodal in 44 cases.

### Survival outcomes for different treatments

**Figure 1:**
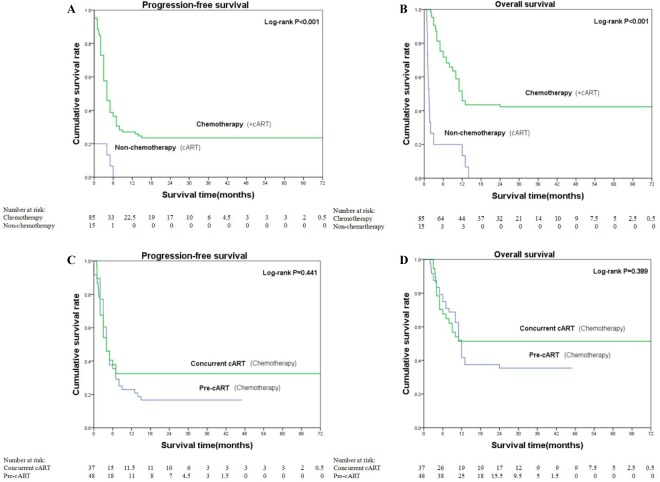
Kaplan-Meier plots comparing OS and PFS for patients receiving different treatment regimens.

The relationship between treatment type and survival is shown in Table 2 and Fig. 1. Among the 100 patients, the 2-year OS rate was 35.0%, and the median PFS duration was 4 months. Objective remission and CR were observed in 44 and 20 patients, respectively.

**Table 2:** The influence of PFS and OS on ARL patients under different treatment regimens.

Variables	All patients (n = 100)	Non-chemotherapy^c^ (n = 15)	Chemotherapy^d^ (n = 85)	P value	Pre-cART (n = 48)	concurrent cART (n = 37)	P value
PFS, median (95% CI)	4.0 (3.2–4.8)	0.0	4.0 (2.9–5.1)	<0.001^a^	4.0 (2.8–5.2)	4.0 (2.2–5.8)	0.441^a^
2-year OS, %(95% CI)	35.0 (25.2–44.8)	0.0	41.0 (29.2–52.8)	<0.001^a^	34.0 (20.3–47.7)	51.0 (35.3–66.7)	0.389^a^
Objective remission rate, n (95% CI)	44.0 (44.0%)	3.0 (20%)	41.0 (48.2%)	0.051^b^	19.0 (39.6%)	22.0 (59.5%)	0.083^b^
Complete Response rate, n (95% CI)	20.0 (20.0%)	0.0	20.0 (23.5%)	0.037^b^	8.0 (16.7%)	12.0 (32.4%)	0.122^b^

^a^Based on log-rank test.

^b^Based on Fisher’s exact test.

^c^Including patients who did not received any treatment and who only received cART treatment.

^d^Patients who received chemotherapy combined with cART regimen.

OS, overall survival; PFS, Progression-free survival.

The 2-year OS rate (41.0% vs. 0%, P < 0.001), median PFS duration (4.0 vs. 0 months, P < 0.001), and CR rate (20.0% vs. 0%,P = 0.037) were significantly higher in patients who received chemotherapy, compared to those who did not. Chemotherapy had no effect on the ORR (P = 0.051), and there were no significant differences in any treatment outcomes between the prior cART and concurrent cART groups (all P values > 0.05).

### Prognostic factors for PFS and 2-year OS in patients with ARL

Table 3 shows the results of our multivariate analysis. Significant predictors of a worse OS included an age <45 years (hazard ratio [HR] 0.45, 95% confidence interval [CI] 0.20–0.97, P = 0.043), high aaIPI (HR 11.92, 95% CI 2.76–51.52, P = 0.001), and high LDH level (HR 2.32, 95% CI 1.09–4.92, P = 0.029). Chemotherapy was a significant positive predictor of OS (HR 0.16, 95% CI 0.06–0.39, P < 0.001) and PFS (HR 0.24, 95% CI 0.09–0.62, P = 0.003). Compared to DLBCL, BL/BLL correlated more unfavorably with OS (HR 5.94, 95% CI 2.49–14.17, P < 0.001) and PFS (HR 2.68, 95% CI 1.20–6.00, P = 0.016). The Ann Arbor stage was not associated with prognosis.

**Table 3:** Multivariate Cox hazard regression for risk factors of OS and PFS in Patients with ARL.

Variables	Hazard ratio (95% CI)			
	OS	P value	PFS	P value
**Age**				
<45 years	Reference		Reference	
≥45 years	0.45 (0.20–0.97)	0.043	1.12 (0.56–2.24)	0.740
**Sex**				
Male	Reference		Reference	
Female	1.49 (0.74–3.00)	0.269	1.10 (0.61–1.97)	0.752
**Baseline CD4 count**				
<100 cells/mm^3^	Reference		Reference	
≥100 cells/mm^3^	0.52 (0.27–1.02)	0.056	0.57 (0.31–1.04)	0.067
**Treatment**				
Non-chemotherapy	Reference		Reference	
Chemotherapy	0.16 (0.06–0.39)	<0.001	0.24 (0.09–0.62)	0.003
**Age-adjusted IPI**				
1–2	Reference		Reference	
3–4	11.92 (2.76–51.52)	0.001	2.14 (0.78–5.89)	0.140
**Ann Arbor stage**				
I–II	Reference		Reference	
III–IV	2.00 (0.99–4.06)	0.054	1.53 (0.80–2.93)	0.201
**LDH**				
Normal LDH	Reference		Reference	
Elevated LDH	2.32 (1.09–4.92)	0.029	1.46 (0.81–2.62)	0.208
**Site**				
Intranodal	Reference		Reference	
Extranodal	1.47 (0.80–2.71)	0.214	1.09 (0.65–1.82)	0.743
**Histology**				
Diffuse large B-cell	Reference		Reference	
Burkitt/Burkitt-like	5.94 (2.49–14.17)	<0.001	2.68 (1.20–6.00)	0.016
Other non-Hodgkin’s lymphoma	0.55 (0.25–1.22)	0.141	0.62 (0.29–1.34)	0.222
Hodgkin’s lymphoma	0.00	0.974	1.04 (0.34–3.25)	0.940

IPI, International Prognostic Index; LDH, lactate dehydrogenase; PFS, progression-free survival; OS, overall survival.

## Discussion

Our retrospective study represents the first systematic evaluation of the clinical characteristics, efficacy, outcomes of chemotherapy plus cART, and multivariate analysis of prognosis of 100 patients with AIDS-related lymphoma in China.

In both our study and another recent study^21^, DLBCL was the most common diagnosis in ARL patients (66% and 69%, respectively). However, the percentage of patients with BL/BLL was lower in our study (12%) than in the previous study (26%), which may reflect the greater incidence of BL/BLL in Europe and the US, compared to Asia^22^. The DLBCL subtype of ARL frequently manifests with characteristics associated with a poor prognosis, including advanced stage, extranodal involvement^10^, a high aaIPI, and a high LDH level^21^. These characteristics were observed in 23%, 49%, 61%, and 73% of the DLBCL patients in our study, respectively, and the latter two were identified as the high-risk characteristics of DLBCLs in our study. Burkitt lymphoma is a highly invasive type of NHL, and the recurrence and metastasis of this tumor type are associated with a significant reduction in the likelihood of survival^23^. Consistent with that observation, the 12 BL/BLL patients in our study had a high aaIPI (100%), elevated LDH level (75%), extranodal involvement (50%), and late-stage disease (75%).

In our study, chemotherapy combined with cART was efficacious and yielded good survival results. Notably, the survival outcomes (2-year OS, PFS, ORR, and CR) did not differ significantly between ARL patients who received cART before or concurrently with chemotherapy. A similar finding was reported in a retrospective study^24^. Intensive chemotherapy may expose patients to opportunistic infections by reducing CD4^+^ cell counts. Although we did not monitor changes in CD4^+^ cell counts, no serious opportunistic infections were observed after chemotherapy. A prospective trial found that CD4^+^ counts declined during chemotherapy, with or without cART^25^. Although the counts rebounded 6 months after chemotherapy to exceed the baseline values in both treatment groups, they remained significantly higher in the cART group.

Furthermore, cART significantly reduced the incidence and improved the prognosis of AIDS-associated NHL^26^. Prior to the advent of cART, patients with this disease usually received low-dose chemotherapy and had a 2-year OS rate of approximately 10%. In our study, patients receiving combined chemotherapy/cART achieved a 2-year OS rate of 41%. We therefore recommend the use of this combined measurement for treatment of ARL, as does the British HIV Association, which based its recommendation on the results of a meta-analysis^27^.

We used multivariate analysis to identify factors that would significantly predict a worse OS in patients with ARL, including a high aaIPI, high LDH level, BL/BLL subtype, age  <45 years, and no chemotherapy. The aaIPI appears to be an important prognostic factor. Consistent with our findings, a previous pooled analysis demonstrated that increases in the aaIPI (range 0–3) correlated strongly with decreases in the survival rate^21^. However, in the pooled analysis, an older (rather than younger) age was a negative predictor of OS; hence, further research with large sample sizes is needed. In a multivariate analysis of patients with AIDS-associated NHL, the clinical stage affected PFS, whereas age, IPI, B symptoms, and hemoglobin levels did not^28^. Researchers in the US established a new prognosis scoring system (i.e., ARL IPI) by combining the HIV scores (prior history of AIDS, baseline CD4^+^ count, and viral load) with lymphoma prognostic factors and found that this system significantly better predicted the risk of death, compared to aaIPI^29^. Although the ARL IPI is not used in China, our study results may provide a foundation for a similar system that would provide a better strategy for clinical treatment and risk assessment.

Antiretroviral therapy should be initiated as soon as possible in patients diagnosed with AIDS, regardless of the CD4^+^ cell count, as the delayed initiation of cART has been shown to significantly increase the risk of developing ARL^30^. This and other studies highlight the importance of cART for the early prevention of lymphoma in AIDS patients. We recommend combined chemotherapy/cART for patients with ARL who cannot use rituximab because of CD20 negativity, treatment expense, lymphoma subtype, or other reasons. cART should be the preferred treatment for ARL patients who cannot receive chemotherapy. Some ARL patients in our study achieved a CR even though they had received cART but not full-course chemotherapy, and other researchers described a patient who achieved a 4-year CR after cART alone^31^. Therefore, cART may play an important role in the treatment of ARL. The importance of chemotherapy, however, should not be overlooked, as the non-chemotherapy group in our study had a 2-year OS of 0%.

Our study had some limitations of note. For example, we did not determine the optimal timing of cART and chemotherapy. This issue requires further study. Furthermore, our observed OS was substantially worse than that reported by a British study^10^, which may be related to a poor baseline performance status or patient choice.

In conclusion, we have shown that ARL patients had better survival outcomes when treated with standard-dose chemotherapy plus cART, compared to cART alone. A high aaIPI, elevated LDH level, BL/BLL pathological subtype, age  <45 years, and non-chemotherapy were indicators of poor prognosis. Our study provides important information for the establishment of ARL guidelines in the future.

## Methods

### Ethical considerations

This retrospective study was approved by the institutional review boards of Henan Provincial Infectious Disease Hospital and The First People’s Hospital of Zhengzhou. The study protocols complied with the principles of the Declaration of Helsinki. The requirement for written informed consent was waived by the institutional review board. Anonymized clinical and therapeutic data were abstracted from electronic medical records at Henan Provincial Infectious Disease Hospital.

### Data collection

The study was conducted at the Henan Provincial Infectious Disease Hospital in China. We collected all patients (performance status 0–2) with a history of AIDS who were newly diagnosed with lymphoma.

### Risk factors

For our analysis, we collected inpatient and follow-up data from 100 patients and assessed the prognostic value of the following factors: sex, age, histological subtype (DLBCL, BL/BLL, and Hodgkin lymphoma, and others), Ann Arbor stage, LDH level (normal vs. elevated), primary site (extranodal vs. intranodal), baseline CD4^+^ count (<100 vs. ≥100 cells/mm^3^), treatment (chemotherapy vs. non-chemotherapy; all chemotherapy regimens used standard doses recommended by the lymphoma guidelines), and age-adjusted IPI (aaIPI). Baseline T cell counts and LDH levels were measured before chemotherapy.

### Study endpoints

For patients who died in hospital, we obtained the date of death from death records. For those patients still alive or died after discharge, we used the date of the last follow-up. Data of patients who remained alive from June 2012 to June 2018 were categorized as truncated data. The major endpoints of this study were progression-free survival (PFS) and OS. PFS is defined as the time from the first day of treatment to the time of disease progression. OS is defined as the time from the initiation of treatment to death for any reason.

### Statistical analysis

Continuous and categorical variables are presented as means ± standard deviations and as numbers and percentages, respectively. Fisher’s exact test was used to evaluate the objective remission rates (ORRs) and CR rates. Multivariate Cox proportional hazards regression analyses were performed to identify potential risk factors for mortality. Kaplan–Meier curves with log-rank tests were used to evaluate differences in OS and PFS between patients in the prior cART and concurrent cART groups. Statistical significance was set at < 0.05. Statistical analyses were performed using IBM SPSS statistics software, version 23.0 (IBM, Armonk, NY, USA).

## Data Availability

All data in the study are available on request.
